# Sabyasachi (Sab) Bhaumik, OBE, MD, DPM, FRCPsych (Hon)

**DOI:** 10.1192/bjb.2020.58

**Published:** 2020-10

**Authors:** Regi Alexander, Sheila Hollins

**Formerly Medical Director, Leicestershire Partnership NHS Trust, and Honorary Chair in Psychiatry, Department of Health Sciences, University of Leicester, UK**

**Figure:**
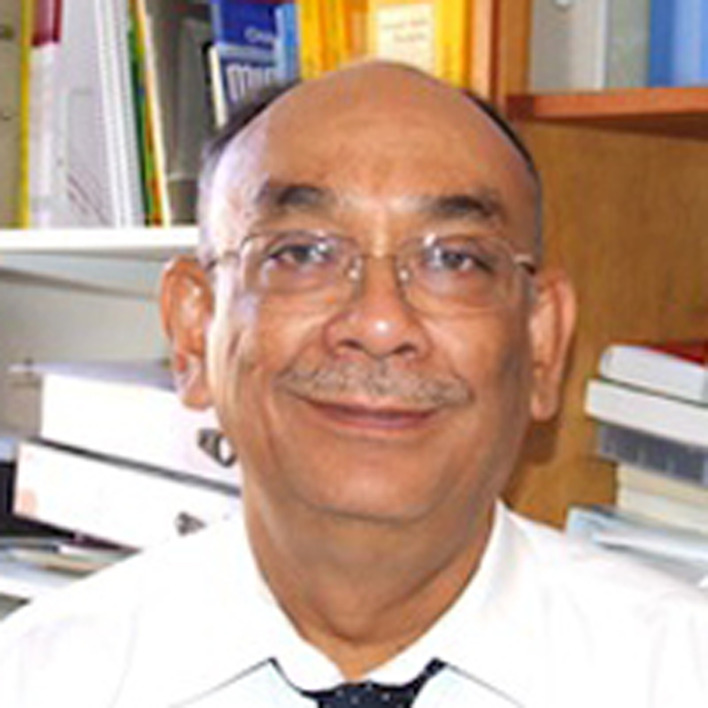

Sabyasachi (Sab) Bhaumik, who died of a massive cardiac infarction at the age of 66 years on 8 November 2019, was a leading figure nationally and internationally in the psychiatry of people with developmental disabilities. He served as Chair of the Faculty of the Psychiatry of Intellectual Disability at the Royal College of Psychiatrists (RCPsych) between 2006 and 2010, and before that as Chair of the Trent Division. In 2007, he established an international links group that brought together all the activities related to intellectual disability undertaken internationally by RCPsych members. Members of this committee have undertaken numerous educational, research and service development projects in India, Pakistan, Sri Lanka, Sudan, Egypt and other parts of Africa and East European countries. More recently he had taken on the role of the World Psychiatric Association's Intellectual Disability Taskforce Lead. On the national scene he was appointed an expert advisor to both the National Institute for Health Research and the National Institute for Health and Care Excellence.

Having recognised a real gap in the body of knowledge on the use of prescribed medications with people with intellectual disabilities, he and David Branford developed the *Frith Prescribing Guidelines for People with Intellectual Disability* (now in its third edition)^1^ and started the annual conference on Therapeutics in Intellectual Disabilities, now in its 11th year. He published over 100 articles and book chapters in his field on a wide range of subjects, including the development of a competency-based framework in intellectual disability psychiatry, the relationship between autism spectrum disorder and visual impairment in individuals with intellectual disabilities, and health promotion in people with intellectual disabilities. Most recently he had (with R.A.) edited the *Oxford Textbook of the Psychiatry of Intellectual Disability*^2^ and, with others, was in the process of editing a textbook on the psychiatry of intellectual disability across cultures.

He had a leadership role among UK psychiatrists belonging to ethnic minorities and served as President of the British Indian Psychiatric Association and Chair of the RCPsych's Diaspora Groups Committee. He spent a considerable amount of time abroad on visits to India training doctors in the psychiatry of intellectual disability and, in recognition of his academic contribution, was appointed Visiting Professor of Psychiatry at SRM University, Chennai, and the Father Muller Medical College, Mangalore, India.

Sab was born in Calcutta in 1952 to Gopal Bhaumik, a well-known Bengali poet, and Uma Bhaumik. After primary and secondary education in Calcutta, he graduated in medicine from R.G. Kar Medical College, Calcutta, in 1978. He then went to work in Khatra, a remote rural area. Equipment, medication and infrastructure were lacking but Sab made up for these with his commitment, compassion and honesty. When he left after 3 years to join an MD programme in pharmacology at Benares Hindu University, there were at least 200 people there waiting to see him off. Moving to the UK in 1985, Sab worked first on a psychiatric rotation in north Wales. He joined the Leicester Frith Hospital as a consultant in intellectual disability in 1992. Subsequently, he was appointed to various senior roles at the hospital – lead clinician, clinical director and medical director. After his retirement in 2013, he worked as a consultant psychiatrist and senior medical advisor to the hospital's board.

Sab was a man of extraordinary energy, generosity and kindness. He was always a powerful advocate for his patients, whom he saw as a marginalised group of people with developmental disabilities who often had neither equity of access nor equity of treatment. His Saturday mornings were spent at the hospital surrounded by people wanting his help and advice – psychiatrists preparing for the RCPsych membership examinations, registrars getting ready for consultant interviews and consultants seeking guidance on a range of assorted issues.

In 2005, he was awarded the Hospital Doctor of the Year award at the London Hilton on Park Lane, where his colleagues spoke warmly of his multidisciplinary approach. In 2006, his enormous contribution was recognised with an OBE for services to medicine. In 2012, he was appointed to an Honorary Chair in Psychiatry in the Department of Health Sciences at Leicester University, a reflection of his contribution to research and teaching. In 2015, the RCPsych awarded him its highest honour – an honorary fellowship.

Soon after his unexpected demise, his wife Susmita Hoare wrote eloquently about how Sab had a ‘magnetic quality to attract people’ and how ‘his honesty, compassion and ability to rise above petty jealousies made him one of a kind’. She spoke for many when she said, ‘Like a comet he blazed into our lives, touching everyone with love, laughter and hope – the world darkening as he left’.

Sab Bhaumik is survived by Susmita and his son Sugato, a junior doctor.
